# Additional Draft Genome Sequences of *Escherichia coli* Strains Isolated from Septic Patients

**DOI:** 10.1128/genomeA.01614-15

**Published:** 2016-01-21

**Authors:** David A. Coil, Guillaume Jospin, Jonathan A. Eisen, Jason Y. Adams

**Affiliations:** a University of California Davis Genome Center, Davis, California, USA; b University of California Davis, Department of Evolution and Ecology, Department of Medical Microbiology and Immunology, Davis, California, USA; c University of California Davis School of Medicine, Department of Internal Medicine, Division of Pulmonary, Critical Care, and Sleep Medicine, Sacramento, California, USA

## Abstract

We present the draft genome sequences of eight uropathogenic strains of *Escherichia coli* isolated from blood cultures collected from patients with sepsis, an extension of previous sequencing work from the same cohort.

## GENOME ANNOUNCEMENT

Previously we reported on the genomes of several uropathogenic *Escherichia coli* isolated from the blood of patients admitted to the UC Davis Medical Center with sepsis from a urinary tract source of infection (1). Here we report on the genomes of an additional eight strains isolated from unique patients in this study. All aspects of the study were approved by the institutional review board of the UC Davis Medical Center (Protocol #247849).

Sepsis is the costliest reason for hospitalization in the United States, affecting more than 1.6 million Americans each year (2, 3). Severe sepsis and septic shock carry a high risk of in-hospital mortality (4, 5) but clinical outcomes vary widely across individuals. One goal of this sequencing effort is to begin to tease out the relative contributions of pathogen genomic variability and host susceptibility factors to clinical outcomes.

Strains of *E. coli* were isolated from overnight plated subcultures of initial liquid blood cultures obtained in the course of routine clinical practice and stored in an −80°C biorepository. A single colony from each strain was grown in LB broth at 37°C, then used for genomic DNA extraction. Illumina paired-end libraries were made from *E. coli* genomic DNA extracted using an Mo Bio Powersoil DNA extraction kit (Mo Bio, Carlsbad, CA). Libraries were prepared using an Illumina TruSeq Kit (Illumina, San Diego, CA). The samples were pooled together and then sequenced on an Illumina MiSeq for paired-end 250-bp reads.

An average of 4,042,576 paired-end reads per sample were generated. Quality trimming and error correction resulted in an average of 3,838,940 high-quality reads. All sequence processing and assembly of the Illumina reads were performed using the A5 assembly pipeline (6). Automated annotation was performed using the RAST annotation server (7). The assembly and annotation statistics are presented in Table 1.

**Table 1:** Accession numbers and assembly statistics for 8 *E. coli* strains

Strain ID	No. of contigs	*N*_50_ contigs (bp)	Total size (bp)	Coverage (×)	% G+C	No. of ORFs^a^	No. of RNAs	Accession no.
JA18	203	132,818	5,476,177	114.4	51	5,522	113	LJXY00000000
JA30	82	405,137	5,123,245	104.8	50	5,019	96	LJYA00000000
JA19	64	435,685	5,206,086	118.8	51	5,127	101	LJXZ00000000
JA33	121	181,292	5,483,875	54.4	51	5,555	99	LJYB00000000
JA37	193	126,070	5,444,516	84.9	51	5,624	120	LJYC00000000
JA38	121	558,486	5,181,476	467.8	51	5,129	109	LJYD00000000
JA16	84	203,917	5,064,344	44.6	51	5,014	105	LJXX00000000
JA9	111	219,137	5,197,856	256.3	50	5,124	105	LJXW00000000

Avg	122	282,818	5,272,197	156	51	5,264	106	

^a^ ORFs, open reading frames.

### Nucleotide sequence accession numbers.

All 8 assemblies described in this paper have been deposited as whole-genome shotgun projects in DDBJ/EMBL/GenBank under the accession numbers provided in Table 1.
